# “On the Margins and Not the Mainstream:” Case Selection for the Implementation of Community Based Primary Health Care in Canada and New Zealand

**DOI:** 10.5334/ijic.2501

**Published:** 2017-06-27

**Authors:** Kerry Kuluski, Nicolette Sheridan, Tim Kenealy, Mylaine Breton, Ann McKillop, Jay Shaw, Jason Xin Nie, Ross EG Upshur, G Ross Baker, Walter P Wodchis

**Affiliations:** 1 Sinai Health System and University of Toronto, CA; 2 University of Auckland, NZ; 3 Université de Sherbrooke, CA; 4 Women’s College Hospital, CA; 5 Sinai Health System, CA; 6 University of Toronto, CA

**Keywords:** primary care, integrated care, aging, Canada, New Zealand, case study

## Abstract

Healthcare system reforms are pushing beyond primary care to more holistic, integrated models of community based primary health care (CBPHC) to better meet the needs of aging populations and their carers. Across the world CBPHC is at varying stages of evolution and no standard model exists. In order to scale up and spread successful models of care it is important to study what works well and why to support broader efforts to implement, scale-up and spread promising innovations. The first step in this endevour is to select appropriate cases to study. In this paper we share our adaptation of case study methodology to iteratively select models of CBPHC in three jurisdictions: Ontario, Quebec (Canada) and New Zealand. A combination of literataure searches (of empirical and gray sources) and stakeholder engagement enabled the selection of cases to study, with the latter providing the most fruitful method. We conclude that it is possible to use personal networks and experts exclusively. It is not clear how much value formal searching adds over and above expert advice. However in a situation where there is no existing definitive list of potential cases, and no acknowledged “gold standard” way to create such a list, it seems appropriate to gather cases using multiple methods and to document those methods systematically.

## Introduction

Aging populations, growing complexity in consumer care needs, and increasing dissatisfaction with poorly integrated health care services are common challenges across health care systems [1,2]. Efforts to improve and integrate health systems gained momentum in the 1980s with a shift from “pure” organizational forms to those with increasingly blurred boundaries between organizations and sectors [3]. We see such a shift in the primary care sector where there are efforts to span the boundaries of “doctors’ workshops” into sectors such as community care, housing, and hospital care, to better serve aging populations and their networks/families. The primary care sector is intended to be the “entry point” into the health system and a foundational component for integrated health care [4]; nevertheless, attempts to reform the primary care sector have been slow and piecemeal [5,6].

Across developed countries primary care reforms have included a movement toward team based care (particularly leveraging the roles of nurses), fee schedule changes (from fee-for-service to mixed/capitation models), and the adoption and sophistication of electronic health records [6,7,8]. Emerging models of CBPHC have extended to link primary care to care in the community – homecare, housing, transportation, recreation, nutritional support, and carer support – to promote health, enhance social connectivity and address cultural, linguistic and spiritual needs [9]. CBPHC in this context has the potential to respond to populations with high needs such as those with compounding jeopardy from chronic conditions, poverty, minority status and age [10]. Integration of primary care with broader health and social sectors aligns with the Alma Alta Declaration of 1978, which asserted that intersectoral approaches to health, community participation and a commitment to equity were essential to achieve primary health care that is promotive, preventive, curative, and inclusive of supportive and rehabilitative services [11]. While some primary care models reflect these principles in their vision and scope [12,13] they are the exception, not the rule.

Most countries seek better models of CBPHC to address the challenges they face in improving the system of care. Our international, interdisciplinary research team of clinicians, scientists, knowledge users, and consumers in Canada (Ontario and Quebec) and New Zealand have collaborated to identify models that address the health and social needs of older adults with complex conditions and their carers. Our purpose is to identify the steps required to scale up and spread successful models, elsewhere.

In this commentary, we describe our methods to select CBPHC models by identifying four key lessons. We reveal the limited utility of sourcing the empirical literature; the difficulty in identifying “successful” models to study when outcomes of importance differ across stakeholders; the value of drawing on clinical and organisational networks and experts; and the association between policy context and ease of case selection. Such insights have important implications for case study methodology in health services and policy research.

## Moving Beyond Primary Care to Primary Health Care

Our team unanimously agreed to select models of CBPHC that demonstrated innovation [14] and sought to avoid conventional and well-researched primary care models. The search criteria included: collaboration between primary care and one or more of: home and community care, secondary/specialist or tertiary provider; providing care to a geographically defined population or network of providers; being person-focused, rather than focusing on a specific disease or condition; and include care for older adults with complex health and social needs.

### Lesson 1: Searching the empirical literature had limited utility

Initial broad searches in electronic databases sought to identify models of care that met our aforementioned inclusion criteria. We found few examples, despite an extensive literature on existing models already known to the researchers. Our search revealed mostly single disease focused interventions (e.g., heart failure) [15], within a single sector/organization (e.g., primary care) with “add ons” such as additional staff employed as navigators to enhance information sharing systems. While these elements are important in linking the disparate parts of health and social care systems, they were considered partial beyond the status quo. We noted that the syntheses of integrated models, including CBPHC, reported in the literature was heavily weighted to randomized controlled trials [15]. A recent Cochrane review on primary care interventions for patients with multimorbidity limited the search to randomized controlled trials, controlled clinical trials, controlled before and after studies, and interrupted time series analyses – and revealed little evidence [16,17]. This literature may have overlooked valuable insights from models that had not been formally evaluated or studies that had not used “gold standard” methods.

### Lesson 2: It is difficult to identify “successful” models when outcomes of importance differ across stakeholders

We also sought to prioritize *successful models* of CBPHC. Our quest to find successful models was complicated by different outcome measures to determine effectiveness. Older patients commonly prioritize functional independence [18] [19], staying connected to their communities and support networks [20] and having a good interpersonal relationship with their care team. Outcomes in health services and policy research prioritize: reduced hospital and emergency services use, reductions in mortality and morbidity, and cost effectiveness. From an economic perspective, an increase in costs from the integration of health and social care resources into communities, despite improved care and quality of life outcomes for patients and their carers, might be judged a “less successful” overall outcome; and financial outcomes appear to carry greater weight in policy contexts.

### Lesson 3: Accessing “informal” sources for case selection was important

Drawing on knowledge from key stakeholders proved a useful strategy and was supplemented, in some jurisdictions, by methodical searching of grey literature. Liaising with government agencies responsible for intersectoral relationships between health and social services informed policy contexts and identified potential interviewees with specific knowledge related to the cases studied. In New Zealand, for example, the Ministry for Māori Development and Whānau Ora takes an interagency approach to health and social care and is mandated to build the capacity of families – this source was integral to case selection. We identified decision-makers and researchers with comprehensive knowledge of integrated networks and the implementation of successful innovations, and who as ‘insiders’, offered historical understanding and context, which could not be identified in the empirical or gray literature. In Quebec, researchers met face-to-face with decision-makers at the provincial level to identify potential exemplar cases for study. Cases were then reviewed by a researcher who had extensive experience researching the implementation of successful innovations within integrated networks for seniors in Quebec. Similarly, the Ontario researchers collaborated with a network that encourages collaborative, community-driven research and knowledge translation in home and community care through the Co-Chair of the Canadian Research Network for Care in the Community. Working with stakeholders informed case selection and supported access to resources required to conduct the case studies.

### Lesson 4: Ease of case study selection varied by policy context

Seawright and Gerring [21] outline techniques of case study selection by accessing different “types” of cases that can be studied. A “typical case”, for example, may be selected to represent the broader population or phenomena under study. A “diverse case” may be selected for maximum variation in a sample, while an “extreme case” (at the margins) might be selected to identify interesting, different or unexpected characteristics. Selecting diverse or extreme cases can strengthen or challenge hypotheses and associations between variables and identify rich contextual data, beyond that usually identified in a “typical” case [22]. Case selection was complicated by the different ways health systems were organized in Ontario, Quebec and New Zealand. Models of CBPHC were identified at the sub-national level – provincially in Canada and through district health boards in New Zealand – where a patchwork of primary health care models were at different stages of development. Quebec, was the exception, and had a more uniform policy structure of local health and social networks, a product of the 2004 government mandate to improve care and well-being for the population within each local territory [23]. The Quebec team chose to vary the cases by local characteristics based on the hypothesis that the complexity of local health and social networks is associated with population density and the number of providers and organizations involved. Given the greater variation of CBPHC in Ontario and New Zealand, case selection was determined incrementally and adapted conventional case study selection approaches.

## Overall Lessons Learned and Implications for Research

Our experience highlights the value of leveraging the expertise of key stakeholders – their connections and “insider” knowledge – which provided us with opportunities to identify models of CBPHC on “the margins”, not found in the mainstream empirical or grey literatures.

We adapted traditional case study selection methods to be more iterative and rigorously searched for gray literature. High value was placed on key stakeholder expertise and our collective knowledge as clinicians and researchers. This was particularly imperative given that the ‘universe’ of available cases from which to select were unclear, particularly in Ontario and New Zealand. Applying a pragmatic rationality, we selected cases across three health care systems that had the potential to deliver optimal CBPHC to older adults with complex needs and their carers. We sought diversity in the populations each case served, which included indigenous, minority and marginalized populations (e.g., Māori and Chinese). We challenged ourselves to go beyond the *mainstream* of evaluated research (empirical literature) to *the margins* where we selected our cases. Finding our cases beyond empirical, evaluated sources suggests a need for the concept of evidence in the context of innovation to be more expansive. For researchers who seek new knowledge through emerging phenomena we offer insights into case selection from our experiences undertaking real-world research.

## References

[1] Advisory Panel on Healthcare Innovation (2015). Unleasing Innovation: Excellent Healthcare for Canada. Report of the Advisory Panel on Healthcare Innovation.

[2] Barnett K, Mercer SW, Norbury M, Watt G, Wyke S, Guthrie B (2012). Epidemiology of multimorbidity and implications for health care, research, and medical education: a cross-sectional study. Lancet.

[3] Denis J-L, Ferlie E, VG N (2015). Understanding hybridity in public organizations. Public Administration.

[4] Starfield B (1998). Primary Care: Balancing Health Needs, Services and Technology.

[5] Hutchison B, Glazier R (2013). Ontario’s Primary Care Reforms Have Transformed The Local Care Landscape, But A Plan Is Needed For Ongoing Improvement. Health affairs.

[6] Hutchison B (2013). Reforming Canadian primary care – don’t stop half-way. Healthc Policy.

[7] Hutchison B, Glazier R (2013). Ontario’s primary care reforms have transformed the local care landscape, but a plan is needed for ongoing improvement. Health Aff (Millwood).

[8] Hutchison B, Levesque JF, Strumpf E, Coyle N (2011). Primary health care in Canada: systems in motion. Milbank Q.

[9] Government of Ontario (2015). Community hubs in Ontario: A strategic framework and action plan Toronto. https://www.ontario.ca/page/community-hubs-ontario-strategic-framework-and-action-plan.

[10] Sheridan NF, Kenealy TW, Kidd JD, Schmidt-Busby JI, Hand JE, Raphael DL (2015). Patients’ engagement in primary care: powerlessness and compounding jeopardy. A qualitative study. Health Expect.

[11] World Health Organization (1978). Declaration of Alma-Ata Alma-Ata. http://www.who.int/publications/almaata_declaration_en.pdf.

[12] Kane RL, Homyak P, Bershadsky B, Flood S (2006). Variations on a theme called PACE. J Gerontol A Biol Sci Med Sci.

[13] Goodwin N, Dixon A, Anderson G, Wodchis W (2014). Providing integrated care for older people with complex needs: lessons from seven international case studies. http://cdn.basw.co.uk/upload/basw_102418-7.pdf.

[14] Greenhalgh T, Robert G, Macfarlane F, Bate P, Kyriakidou O (2004). Diffusion of innovations in service organizations: systematic review and recommendations. Milbank Q.

[15] Boult C, Green AF, Boult LB, Pacala JT, Snyder C, Leff B (2009). Successful models of comprehensive care for older adults with chronic conditions: evidence for the Institute of Medicine’s “retooling for an aging America” report. J Am Geriatr Soc.

[16] Smith SM, Soubhi H, Fortin M, Hudon C, O’Dowd T (2012). Interventions for improving outcomes in patients with multimorbidity in primary care and community settings. Cochrane Database Syst Rev.

[17] Smith SM, Soubhi H, Fortin M, Hudon C, O’Dowd T (2012). Managing patients with multimorbidity: systematic review of interventions in primary care and community settings. BMJ.

[18] Fried TR, Tinetti ME, Iannone L, O’Leary JR, Towle V, Van Ness PH (2011). Health outcome prioritization as a tool for decision making among older persons with multiple chronic conditions. Arch Intern Med.

[19] Kuluski K, Gill A, Naganathan G, Jaakkimainen R, Wodchis WP (2013). A qualitative descriptive study on the alignment of care goals between older persons with multi-morbidities, their family physicians and informal caregivers. BMC Family Practice.

[20] Naganathan G, Kuluski K, Gill A, Jaakkimainen L, Upshur R, Wodchis WP (2016). Perceived value of support for older adults coping with multi-morbidity: patient, informal care-giver and family physician perspectives. Ageing & Society.

[21] Seawright J, Gerring J (2008). Case Selection Techniques in Case Study Research: A Menu of Qualitative and Quantitative Options. Political Research Quarterly.

[22] Flyvbjerg B (2006). Five Misunderstandings About Case-Study Research. Qualitative Inquiry.

[23] Hebert R, Durand PJ, Dubuc N, Tourigny A, Group P (2003). PRISMA: a new model of integrated service delivery for the frail older people in Canada. Int J Integr Care.

